# Kidney disease as a determinant of cognitive decline and dementia

**DOI:** 10.1186/s13195-015-0115-4

**Published:** 2015-03-17

**Authors:** Thorleif Etgen

**Affiliations:** Department of Psychiatry and Psychotherapy, Technische Universität München, Klinikum rechts der Isar, Ismaninger Strasse 22, 81675 München, Germany; Department of Neurology, kbo-Inn-Salzach-Klinikum, Gabersee 7, 83512 Wasserburg am Inn, Germany

## Abstract

Chronic kidney disease (CKD) has evolved as a possible new determinant of cognitive decline and dementia. This review outlines the presumed pathophysiology of cognitive decline in CKD, which consists of traditional and new vascular risk factors as well as nonvascular risk factors and metabolic and biochemical abnormalities within the central nervous system caused by CKD. The recent major cross-sectional studies and longitudinal studies – including one meta-analysis – that mostly suggest an association of cognitive decline and CKD are discussed. Finally, potential therapeutic strategies are presented.

## Introduction

Cognitive decline comprising cognitive impairment and dementia is of increasing interest due to a worldwide ageing population with, on the one hand, a rising incidence of cognitive decline and, on the other, limited therapeutic options. The potential for primary prevention of dementia has been thoroughly studied and possibly one-third of Alzheimer’s dementia cases might be attributable to potentially modifiable risk factors [1]. The concept of mild cognitive impairment, which describes cognitive impairment beyond that of normal ageing but in contrast to dementia, does not interfere notably with activities of daily life [2], permits timely identification of patients at high risk of developing dementia and implies the potential of a larger therapeutic window for modifiable risk factors [3]. Among these somatic risk factors, chronic kidney disease (CKD) has been discussed as a potential independent risk factor for cognitive decline [4,5].

The aims of this review are to summarize the anatomical and neuroimaging background and pathophysiology of cognitive decline in CKD, to provide an updated overview of the major clinical studies of the association between CKD and cognitive decline, and to indicate possible therapeutic strategies.

## Anatomical and neuroimaging background

The kidney and the brain possess a similar low vascular resistance system allowing continuous high-volume perfusion, which makes both organs vulnerable to microvascular injury caused by hypertension and diabetes [6]. The resulting small-vessel disease manifests in both organs: in the brain, it leads to white matter lesions that contribute to cognitive decline; and in the kidney, it is characterized by glomerular endothelial dysfunction and lipohyalinosis accounting for CKD [7].

This anatomical analogy is supported by neuroimaging results demonstrating that individuals with lower estimated glomerular filtration rate (eGFR) have a greater volume of white matter lesions, more silent brain infarcts and cerebral microbleeds [7]. According to one recent longitudinal study, however, CKD is associated with dementia even independent of cerebral small-vessel disease [8]. In this Japanese study with 600 older participants, CKD at baseline was associated with an increased risk of all-cause dementia during a mean follow-up of more than 7 years and after adjusting for magnetic resonance imaging findings and confounding variables (hazard ratio 1.96, 95% confidence interval (CI) 1.08 to 3.58). Magnetic resonance imaging findings included cerebral atrophy (medial temporal lobe atrophy or bicaudate ratio indication subcortical atrophy) and small-vessel disease (represented by lacunar infarction or white matter lesions) [8].

## Pathophysiology of cognitive decline in chronic kidney disease

**Figure 1:**
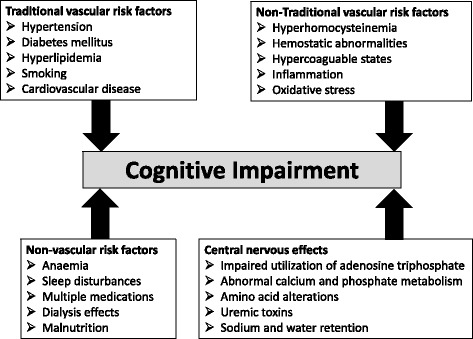
**Pathophysiology of cognitive decline in patients with chronic kidney disease.**

Traditional vascular risk factors such as hypertension, diabetes mellitus, hyperlipidemia, cigarette smoking and cardiovascular disease with myocardial infarction and atrial fibrillation have been linked to cognitive decline in patients with CKD [5] (Figure 1).

Nontraditional vascular risk factors including hyperhomocysteinemia, hemostatic abnormalities or hypercoaguable states, inflammation and oxidative stress are also associated with cognitive impairment in patients with renal failure [4]. Possible mechanisms comprise direct prothrombotic effects on the vascular system leading to large-vessel and small-vessel disease. Furthermore, endothelial dysfunction mediated by hyperhomocysteinemia is associated with ischemic leukoaraiosis [9]. Finally, hyperhomocysteinemia has a direct neurotoxicity through overstimulation of *N*-methyl-d-aspartate receptors [10].

In addition, nonvascular risk factors might further contribute to cognitive decline in patients with CKD. Anemia in CKD has been associated with cognitive impairment, and treatment of anemia showed a progressive effect on improving cognitive function in CKD patients [11]. Multiple medications are required in CKD patients and the optimal dosing of several medications is unclear; these patients are therefore more susceptible to side effects and interactions between medications [5]. Sleep disturbances are frequent in patients with end-stage CKD, resulting in impaired concentration, excessive daytime fatigue and possibly cognitive dysfunction [12].

Finally, multifactorial metabolic and biochemical abnormalities within the central nervous system in renal failure might further account for cognitive impairment. Secondary hyperparathyroidism leading to an increased calcium uptake impairs metabolism of neurotransmitters such as brain gamma-aminobutyric acid, norepinephrine and acetylcholine [13]. Amino acid derangements (mainly glutamine, glycine, aromatic and branched-chain amino acids) leading to subsequent imbalance of neurotransmitters – mainly gamma-aminobutyric acid, dopamine and serotonin – contribute to cognitive impairment [14]. Uremic toxins such as guanidine compounds (creatinine, guanidine, and so forth) also have a neurotoxic effect by activation of *N*-methyl-d-aspartate receptors and concomitant inhibition of gamma-aminobutyric acid receptors [15].

## Overview of major studies

### Cross-sectional studies

The majority of larger cross-sectional studies reported an increased risk of cognitive decline in the presence of CKD. The Heart Estrogen/Progestin Replacement Study examined 1,015 older women with established coronary artery disease and found an approximately 15 to 25% increase in risk for global cognition, executive function, language and memory per 10 ml/minute/1.73 m^2^ decrement in eGFR [16]. The Third National Health and Nutrition Examination Survey observed poorer learning/concentration (odds ratio (OR) 2.4, 95% CI 1.3 to 5.6) and impairment in visual attention (OR 2.7, 95% CI 1.0 to 7.4) in association with moderate CKD (eGFR 30 to 59 ml/minute/1.73 m^2^) among 4,849 young, healthy, ethnically diverse participants [17]. The Reasons for Geographic and Racial Differences in Stroke Study reported for patients with CKD among 23,405 participants that each 10 ml/minute/1.73 m^2^ decrease in eGFR was associated with an 11% increased prevalence of cognitive impairment (OR 1.1, 95% CI 1.0 to 1.2) [18]. The Maine-Syracuse Longitudinal Study noticed among 923 individuals free from dementia that global performance and specific cognitive functions were negatively affected early in CKD [19].

The Chronic Renal Insufficiency Cohort study contributed two analyses. Using six different cognitive tests, the first substudy with 825 participants (mean age 65 years) reported participants with advanced CKD (eGFR <30 ml/minute/1.73 m^2^) were, after multiple adjustment, more likely to have clinically significant cognitive impairment on global cognition (OR 2.0, 95% CI 1.1 to 3.9), naming (OR 1.9, 95% CI 1.0 to 3.3), attention (OR 2.4, 95% CI 1.3 to 4.5), executive function (OR 2.5, 95% CI 1.9 to 4.4) and delayed memory (OR 1.5, 95% CI 0.9 to 2.6) compared with those with mild to moderate CKD (eGFR 45 to 59 ml/minute/1.73 m^2^) [20]. The main Chronic Renal Insufficiency Cohort study found among 3,591 participants a higher prevalence of cognitive impairment in those with lower eGFR (OR 1.5, 95% CI 1.1 to 2.1), independent of traditional vascular risk factors. However, this association was no longer significant after adjustment for hemoglobin concentration [21].

### Longitudinal studies

The majority of recent prospective studies found an association between CKD and cognitive decline (Table 1). The Cardiovascular Health Cognition Study reported that an increase in creatinine from 1.0 to 2.0 mg/dl was associated with a 26% increased risk of vascular-type dementia [22]. The Health, Aging, and Body Composition Study demonstrated that more advanced stages of CKD were associated with an increased risk for cognitive impairment [23]. The German Intervention Project on Cerebrovascular Diseases and Dementia in the Community of Ebersberg study found that moderate-to-severe impaired renal function was associated with incident cognitive impairment after 2 years in a large cohort of older subjects [24]. In the Northern Manhattan Study, decreased kidney function estimated by two different formulas was associated with greater cognitive decline, even in those with mild CKD [25]. The Rush Memory and Aging Project indicated that impaired baseline kidney function was associated with a more rapid rate of cognitive decline, especially in semantic, episodic and working memory, but not in visuospatial abilities or perceptual speed [26]. The Osaki-Tajiri Project from Japan showed CKD to be strongly associated with incident dementia even after multiple adjustments for cardiovascular risk factors including anemia [27]. The Maine-Syracuse Longitudinal Study observed a decline in eGFR values being associated with a decline in global cognitive ability, verbal episodic memory and abstract reasoning over time [28]. The Cardiovascular Health Study demonstrated a higher risk of worsening cognitive function in older adults with lower kidney function [29].

**Table 1:** **Major longitudinal studies about the association of chronic kidney disease and cognitive decline (modified after** [34]**)**

**Study**	**Participants**	**Mean age (years)**	**Follow-up (years)**	**Cognitive test**	**Assessment (1) and classification (2) of renal function**	**Adjustment for confounders**	**Result (risk for cognitive decline depending on renal function/proteinuria/albuminuria)**
Osteoporotic Fractures in Men Study [30]	5,929 men	74	5	3MS, Trails B	(1) MDRD	Age, education, race, health status, ADL impairment, alcohol, diabetes, hypertension, CHD, stroke, BMI, PAD	Not significant for both tests in both CKD groups
					(2) Mild CKD = eGFR 45 to 59, moderate CKD = eGFR <45		
Rancho Bernardo Study [31]	1,345	75	6.6	MMSE, Trails B, Category Fluency Test	(1) MDRD	Age, hypertension, HbA1c, dyslipidemia, education, exercise, alcohol, estrogen, depression	Not significant for eGFR
					(2) Moderate-to-severe CKD = eGFR <60		Significant only for men and baseline albuminuria
Three C Study [32]	7,839	74	7	MMSE	(1) CKD-EPI	Age, sex, education, ApoE, hypertension, CHD, dyslipidemia, diabetes, smoking, BMI, stroke	Not significant except for eGFR decline over first 4 years and vascular dementia
					(2) CKD = eGFR <60		Borderline risk for proteinuria
Reasons for Geographic and Racial Differences in Stroke [33]	19,399	64	3.8	6-Item Screener	(1) CKD-EPI	Age, sex, race, education, region, hypertension, diabetes, stroke, CHD, alcohol, smoking	Not significant for eGFR <60
					(2) CKD = eGFR <60		OR 1.30 (95% CI 1.02 to 1.66) for UACR <10 mg/g in eGFR <60
Cardiovascular Health Cognition Study [22]	3,349	77	6	Cognitive battery testing for dementia similar to DSM-IV criteria	(1) Inverse of creatinine	Age, sex, race, body weight, education, CHD, stroke, hypertension, diabetes, smoking, apoE genotype	37% increased risk of dementia (95% CI 1.06 to 1.78)
					(2) Moderate CKD = SCr ≥1.3 mg/dl for women and ≥1.5 for men		
Health, Aging, and Body Composition Study [23]	3,034	74	2, 4	3MS	(1) MDRD at baseline	Age, sex, race, education, diabetes, medication, hypertension, hyperlipidemia, CRP, interleukin-6, hematocrit, CHD, stroke	OR 1.32 (95% CI 1.03 to 1.69) for eGFR 45 to 59
					(2) CKD = eGFR <60 with two subgroups (eGFR 45 to 59 and <45)		OR 2.43 (95% CI 1.38 to 4.29) for eGFR <45
INVADE study [24]	3,697	68	2	6-Item Cognitive Impairment Test	(1) CG at baseline	Age, sex, smoking, CHD, stroke, hypertension, diabetes, BMI, hyperlipidemia, alcohol, physical activity, depression	Moderate-to severe CKD: OR 2.14 (95% CI 1.18 to 3.87)
					(2) Mild CKD = eGFR 45 to 59, moderate-to-severe CKD = eGFR <45		
Rush and Memory Aging Project [26]	886	81	3.4	Battery of 19 tests with five cognitive systems	(1) MDRD at baseline	Age, sex, education, BMI, hemoglobin, physical activity, social activity, hypertension, diabetes, smoking, CHD, stroke, PAD, depression	Each GFR reduction of 15 = increased rate of global cognitive decline of being 3 years older
					(2) CKD = eGFR <60		
Northern Manhattan Study [25]	2,172	72	2.9	TICS	(1) CG + MDRD at baseline	Age, sex, race, education, insurance, hypertension, diabetes, alcohol, smoking, CHD, stroke homocysteine, hematocrit, psychoactive medication	Decline by 0.3 TICS points/year for eGFR <60
					(2) Mildly reduced renal function = eGFR 60 to 90, eGFR <60		Decline by 0.2 TICS points/year for eGFR = 60 to 90
Osaka-Tajiri Project [27]	497	74	5	Clinical Dementia Rating	(1) Not described	Age, sex, education, hypertension, diabetes, dyslipidemia, CHD, anemia	Conversion to dementia OR 5.3 (95% CI 1.7 to 16.2)
					(2) CKD = eGFR <60 or albuminuria		
Maine-Syracuse Longitudinal Study [28]	590	62	5	Composite scores of VM, VSOM, ST and WM	(1) MDRD	Age, sex, education, race, diabetes, BMI, smoking, HDL cholesterol, hypertension	Global cognitive ability: *b* = 0.21 SD decline/unit ln(eGFR) (95% CI 0.04 to 0.38)
					(2) CKD = eGFR <60		Verbal episodic memory: *b* = 0.28 SD decline per unit ln(eGFR) (95% CI 0.02 to 0.54)
							Abstract reasoning: *b* = 0.36 SD decline per unit ln(eGFR) (95% CI 0.04 to 0.67)
Cardiovascular Health Study [29]	3,907	75	5.3	3MS, DSST	(1) Cystatin C-based eGFR	Age, sex, race, education, smoking, BMI, diabetes, hypertension, CRP, ApoE, depression	Points/year faster decline:
					(2) CKD = eGFR <60		OR 0.64 (95% CI 0.51 to 0.77) in 3MS
							OR 0.42 (95% CI 0.28 to 0.56) in DSST
Osaka Follow-up Study for Carotid Atherosclerosis, Part 2 [8]	600	68	7.5	MMSE	(1) MDRD	Age, sex, ApoE, education, hypertension, diabetes, cerebrovascular events	HR 1.96 (95% CI 1.08 to 3.58)
					(2) CKD = eGFR <60	brain atrophy, SVD	

ADL, activities of daily living; ApoE, apolipoprotein E genotype; BMI, body mass index; CG, Cockcroft–Gault equation; CHD, coronary heart disease; CI, confidence interval; CKD, chronic kidney disease; CKD-EPI, Chronic Kidney Disease Epidemiology Collaboration equation; CRP, C-reactive protein; DSST, Digit Symbol Substitution Test; eGFR, estimated glomerular filtration rate (ml/minute/1.73 m^2^); HDL, high-density lipoprotein; HR, hazard ratio; INVADE, Intervention Project on Cerebrovascular Diseases and Dementia in the Community of Ebersberg; 3MS, Modified Mini-Mental State Examination; MDRD, Modification of Diet in Renal Disease; MMSE, Mini-Mental State Examination; OR, odds ratio; PAD, peripheral artery disease; SCr, serum creatinine; SD, standard deviation; ST, scanning and tracking; SVD, small-vessel disease; TICS, telephone interview for cognitive status; UACR, urine albumin–creatinine ratio; VM, verbal episodic memory; VSOM, visual–spatial organization and memory; WM, working memory.

However, some studies reported nonsignificant or only partial significant results. The Osteoporotic Fractures in Men Study found an independent association between mild-to-moderate impaired renal function and poor executive function at baseline but not with global cognitive impairment or risk of cognitive decline in older men [30]. The Rancho Bernardo study yielded an association between reduced cognitive function at follow-up only for baseline albuminuria and only for men, but neither for women nor for baseline eGFR [31]. In the French Three C study, no increased risk of cognitive decline or dementia was associated with low eGFR level, although faster decline of renal function was associated with global cognitive decline and incident dementia with vascular component [32]. The Reasons for Geographic and Racial Differences in Stroke study reported that an impaired eGFR was not associated independently with cognitive impairment if compared with preserved eGFR unless albuminuria was added to the stratification [33].

### Meta-analysis

One meta-analysis explored the impact of CKD on cognitive decline. Six cross-sectional studies and six longitudinal studies comprising 54,779 participants could be included in this meta-analysis. Concerning cross-sectional studies, meta-analytic pooling using a random-effects model showed that participants with CKD had a significantly increased risk of cognitive impairment compared with those without CKD (OR 1.65, 95% CI 1.32 to 2.05). Among longitudinal studies, participants with CKD had a significantly increased risk of incident cognitive impairment at follow-up compared with those without CKD (OR 1.39, 95% CI 1.15 to 1.68). Both meta-analyses comprised a significant heterogeneity amongst the studies (degrees of freedom = 9, *P* = 0.0005 and degrees of freedom = 11, *P* <0.0001, respectively) [34]. The association of patients with CKD having a significantly increased risk of cognitive impairment compared with those without CKD remained present independent of the stage of CKD, and was even stronger in the group with moderate-to-severe CKD (GFR <45 ml/minute/1.73 m^2^) compared with mild-to-moderate CKD (GFR 45 to 60 ml/minute/1.73 m^2^). Further sensitivity analyses by grouping studies according to various characteristics such as sample size of the study populations, duration of follow-up and method used to evaluate cognitive function yielded no significant differences across studies [34].

### Limitations

As pointed out in recent reviews, methodological problems of both cross-sectional studies and longitudinal studies limit their interpretation and may also explain divergent results [34,35]. Methods for assessing cognitive function showed great variability, ranging from the 6-Item Cognitive Impairment Test [24], 6-Item-Screener [32] or Mini-Mental Status Examination [21,23,32] to a battery of multidomain cognitive tests [16,19,26]. The study population varied from gender specific – for example, women with coronary heart disease [16] or men [30] – to community-dwelling participants [24]. The mean age of the study population ranged from 36 years [17] to 81 years [26]. Different methods of assessing CKD were applied (for example, Modification of Diet in Renal Disease equation, Cockcroft–Gault equation, CKD Epidemiology Collaboration equation, cystatin-based eGFR). The more sensitive method of albuminuria was seldom applied. The extent of potential confounders ranged from only a few confounding factors to many, including recent confounders such as physical activity, depression or apolipoprotein E [35].

## Conclusions and future directions

Data from pathophysiology, anatomy and neuroimaging studies provide substantial background for the hypothesis of an independent association between CKD and cognitive decline, which is further underlined by the results of most cross-sectional and longitudinal studies including one meta-analysis. Therapeutic strategies will account for the different risk factors, but only few intervention trials have been performed. For example, treatment of hyperhomocysteinemia with high daily doses of B vitamins did not affect cognitive outcomes after 1 year in a randomized placebo-controlled trial of 659 advanced CKD patients [36], but it remains unclear whether a substitution in early CKD might be beneficial.

Improving renal anemia by erythropoietin could improve cognitive function, but one double-blind randomized trial of darbepoetin in CKD patients with moderate anemia did not specifically assess cognitive function. Furthermore, darbepoetin did not reduce the major outcome (risk of death, cardiovascular event or renal event) and was associated with an increased risk of stroke, which is a risk factor for dementia [37]. Successful kidney transplantation yielded long-term improvement in the cognitive performance of patients on chronic dialysis [38].

Whether maintaining blood pressure levels lower than current recommendations further reduces the risk of age-related cognitive decline in patients with and without CKD is currently being investigated in one ongoing large trial (Systolic Blood Pressure Intervention Trial) [39]. Further research and intervention strategies are required and will help to explore the association of cognitive decline in CKD.

## Note

This article is part of a series on *The impact of acute and chronic medical disorders on accelerated cognitive decline*, edited by Carol Brayne and Daniel Davis. Other articles in this series can be found at http://alres.com/series/medicaldisorders

## Abbreviations

CI: Confidence interval
CKD: Chronic kidney disease
eGFR: Estimated glomerular filtration rate
OR: Odds ratio
